# Possible posttraumatic stress disorder in Chinese frontline healthcare workers who survived COVID-19 6 months after the COVID-19 outbreak: prevalence, correlates, and symptoms

**DOI:** 10.1038/s41398-021-01503-7

**Published:** 2021-07-05

**Authors:** Li-Juan Xiong, Bao-Liang Zhong, Xiong-Jing Cao, Huang-Guo Xiong, Ming Huang, Jing Ding, Wen-Tian Li, Jun Tong, He-Yong Shen, Jia-Hong Xia, Yu Hu

**Affiliations:** 1 Department of Nosocomial Infection Management, Union Hospital, Tongji Medical College, Huazhong University of Science and Technology, Wuhan, 430022 Hubei China; 2 Affiliated Wuhan Mental Health Center, Tongji Medical College of Huazhong University of Science and Technology, Wuhan, 430012 Hubei province China; 3 State Key Laboratory of Component-Based Chinese Medicine, Tianjin University of Traditional Chinese Medicine, Tianjin, 301617 China; 4 Research Center for Psychological and Health Sciences, China University of Geosciences, Wuhan, 430074 Hubei province China; 5 Institute of Analytical Psychology, City University of Macau, Macau, China; 6 Department of Cardiovascular Surgery, Union Hospital of Tongji Medical College, Huazhong University of Science and Technology, Wuhan, 430022 Hubei China; 7 Institute of Hematology, Union Hospital, Tongji Medical College, Huazhong University of Science and Technology, Wuhan, 430022 Hubei China

**Keywords:** Depression, Scientific community

## Abstract

Suffering from COVID-19 and witnessing the suffering and deaths of patients with COVID-19 may place frontline healthcare workers (HCWs) at particularly high risk for posttraumatic stress disorder (PTSD); however, few data are available on the clinical characteristics of PTSD among frontline HCWs who survived COVID-19 (“surviving HCWs” hereafter). The present study examined the prevalence, correlates, and clinical symptoms of possible PTSD in surviving HCWs 6 months after the COVID-19 outbreak in China. A total of 291 surviving HCWs and 42 age- and gender-matched COVID-19-free frontline HCWs (control group) were recruited and administered the Chinese Essen Trauma Inventory, which was used to assess the presence of possible PTSD according to DSM-IV-TR criteria. Survivors’ clinical data and characteristics of exposure to COVID-19 were collected via self-report questionnaires. Surviving HCWs had significantly higher rates of possible PTSD than controls (19.9% vs. 4.8%, *P* = 0.017). Correlates of PTSD in survivors were ICU admission (OR = 8.73, *P* = 0.003), >10 respiratory symptoms during the most symptomatic period of COVID-19 (OR = 3.08, *P* = 0.006), the residual symptom of dizziness (OR = 2.43, *P* = 0.013), the residual symptom of difficult breathing (OR = 2.23, *P* = 0.027), life in danger due to COVID-19 (OR = 16.59, *P* = 0.006), and exposure to other traumatic events (OR = 2.94, *P* = 0.035). Less commonly seen PTSD symptoms in survivors were having nightmares about the event (34.5%), suddenly feeling like they were living through the event suddenly (25.9%), being unable to remember an important part of the event (32.8%), and overalertness (31.0%). Nearly one-fifth of the surviving HCWs had possible PTSD 6 months after the COVID-19 outbreak. Mental health services for this vulnerable population should include periodic screening for PTSD, expanded social support, and, when necessary, psychotherapy and psychopharmacological treatment.

## Introduction

The ongoing coronavirus disease 2019 (COVID-19) pandemic has been the most severe global public health crisis since the 1918 flu pandemic. Findings from recent studies have shown that posttraumatic stress disorder (PTSD), a common psychological consequence of major disasters and pandemics, has become a substantial mental health challenge for the general population, COVID-19 patients, and healthcare workers (HCWs) [1,2,]. For example, during the COVID-19 pandemic, as many as 96.2% of the COVID-19 patients in China had significant PTSD symptoms, 30.2% of the COVID-19 patients who recovered from COVID-19 in Italy developed PTSD, and 16.7% of the HCWs in Greece met the criteria for a probable PTSD diagnosis [3–5].

Frontline HCWs are the main force in the battle against COVID-19. During the period of exponential increase in the number of COVID-19 cases, frontline HCWs were overwhelmed by COVID-19 patients’ care [6]. Particularly, in the early period of the COVID-19 outbreak in many countries, due to inadequate supplies of protective gear, frontline HCWs had to serve under high risk of direct exposure to severe acute respiratory syndrome coronavirus 2 (SARS-CoV-2) [7]. Therefore, heightened stress reactions such as fatigue, physical and mental exhaustion, fear, anxiety, and even acute stress disorder (ASD) were more likely to manifest in this population [8,9,]. Importantly, frontline HCWs have witnessed the suffering and deaths of patients with COVID-19, which increase their susceptibility to PTSD. Accordingly, studies have reported a significantly higher prevalence of PTSD symptoms and significantly more severe PTSD symptoms in frontline than nonfrontline HCWs [10–12].

Infections and deaths due to COVID-19 in HCWs are a heartbreaking tragedy but are difficult to avoid worldwide [13–15]. In China, as of 24 February 2020, a total of 3387 HCWs were infected with COVID-19 with 23 deaths [16]. In the United Kingdom, from 25 March to 13 May 2020, there were 147 frontline HCWs died due to COVID-19 [17]. Both COVID-19, a life-threatening disease, and certain treatments, such as tracheal intubation, place HCWs at risk for PTSD [18]. As a result, COVID-19 has been found to be significantly associated with an ~2- to 3-fold risk of PTSD symptoms in HCWs [10,19,].

Currently, there are a few studies on PTSD in frontline HCWs [11, 20–22]. In these studies, the prevalence of PTSD symptoms ranged widely, from 16.8 to 62.0%, and factors associated with PTSD symptoms were female gender, a short length of work experience, a low level of job satisfaction, senior technical title (vs. junior and intermediate), nurses (vs. other categories of HCWs), and negative coping styles. However, to our knowledge, few studies have specifically examined PTSD in frontline HCWs who had COVID-19, and, because of this, the relationships between PTSD and factors such as respiratory symptoms and treatments in frontline HCWs remain unknown. PTSD symptoms typically appear within 3 months of the trauma. Moreover, the symptoms may appear later and persist for months and sometimes years [23]. The limitation of these studies is that PTSD assessments were carried out during the acute period of the COVID-19 outbreak and were not able to show the PTSD symptoms of the subjects under post-COVID-19 conditions.

Maintaining and promoting the mental health of HCWs is an essential part of the public health response to COVID-19 worldwide. Frontline HCWs who survived COVID-19 (“surviving HCWs” hereafter) are a vulnerable group in need of mental health services due to their unique experiences of exposure to COVID-19. Detailed empirical data regarding the mental health problems of HCWs might facilitate the planning and implementation of mental health care programs for HCWs. The present study investigated the prevalence, correlates, and symptoms of possible PTSD in surviving Chinese frontline HCWs 6 months after the COVID-19 outbreak.

## Methods

### Participants

In May 2020, after COVID-19 was under control in China, we initiated a municipality-wide mental health care scheme for HCWs who had worked on the frontline during the COVID-19 outbreak in Wuhan. The aim of the scheme was to provide psychosocial support, psychological consultation, psychotherapy, psychiatric treatment, and crisis intervention for frontline HCWs. In July 2020, we invited the over 1000 surviving HCWs covered by the scheme to participate in this study. Finally, 291 survivors voluntarily participated and completed the study questionnaires. Most survivors became infected between late January and early February 2020; thus, the assessment time-point was ~6 months after the outbreak in China. HCWs ≥18 years old who had a history of direct exposure to and a laboratory-confirmed diagnosis of COVID-19 and were recovered at the time of the study were invited to join the study. Medical students who had COVID-19 were excluded. To examine the risk of PTSD in surviving HCWs, we purposefully recruited 42 age- and gender-matched controls. They were frontline HCWs from 2 COVID-19 designated hospitals in Wuhan, and had not been infected with SARS-CoV-2.

The Institutional Review Board of Wuhan Mental Health Center approved the study protocol and all participants signed the informed consent forms.

### Procedures and measures

An anonymous self-administered questionnaire was sent to surviving HCWs and controls, which was completed and submitted online via the platform of the mental health care scheme.

#### Possible PTSD

The validated Chinese version of the Essen Trauma Inventory (ETI) was used to diagnose possible PTSD [24]. The ETI was developed based on DSM-IV-TR diagnostic criteria and has 5 parts with a total of 58 items. Part 1 contains a list of 14 potentially traumatic events and an open question asking about the exposure to other traumatic events. Part 2 has 10 questions regarding the worst event’s objective and subjective threat to life (criteria A1 and A2). Part 3 consists of 23 questions covering 3 symptom clusters of PTSD (intrusion [criteria B], avoidance [criteria C], and hyperarousal [criteria D]) and one additional symptom cluster for ASD (dissociation). Part 4 assesses the severity of psychological distress caused by the event [criteria F] and the duration of PTSD symptoms [criteria E]. Part 5 evaluates the severity of functional impairment caused by the event in terms of life satisfaction, school/work/job performance, household chores and duties, hobbies and leisure activities, relationships with friends, family relationships, and sexual life [criteria F]. According to the DSM-IV-TR criteria, a possible PTSD diagnosis was operationalized as the endorsement of ≥1 question of A1, ≥1 question of A2, a response of “often” or “very often” on ≥1 question of B, a response of “often” or “very often” on ≥3 questions of B, a response of “often” or “very often” on ≥2 questions of C, a response of at least “a month” of E, and a response of at least “moderately” on ≥1 question of F.

#### Demographics

Demographic variables included gender, age, education, years of work experience, and HCW type.

#### Clinical data

We used a standardized form to collect the clinical characteristics of surviving HCWs, including the severity of COVID-19 at its acute phase, the total number of respiratory symptoms during the most symptomatic period of COVID-19, the length of hospital stay, admission to the intensive care unit (ICU), and residual respiratory symptoms at the time of the study. The total number of respiratory symptoms was collected from a checklist of 21 specific respiratory symptoms: fever, chills, dry cough, cough, stuffy nose, runny nose, sore throat, headache, fatigue, joint pain, muscle pain, dizziness, shortness of breath, dyspnea, chest tightness, chest pain, conjunctival congestion, nausea, vomiting, diarrhea, and abdominal pain. Residual respiratory symptoms included headache, dizziness, palpitations, and difficult breathing.

#### Exposure to COVID-19 and other traumatic events

Four yes–no questions were used to assess the severity of exposure to COVID-19: “Were you physically harmed by COVID-19?”, “Did you think your life was in danger due to COVID-19?”, “Was someone else physically harmed by COVID-19?”, and “Did you think someone else’s life was in danger due to COVID-19?”. A person who reported any traumatic event other than COVID-19 in Part 1 of the ETI was considered as having exposure to other traumatic events.

### Statistical analysis

Rates of possible PTSD were calculated for surviving HCWs, controls, and subgroups according to demographic, clinical, and COVID-19 exposure characteristics. The Chi-square test was used to compare rates between/across subgroups. Multiple binary logistic regression with a backward stepwise entry of all significant factors in the Chi-square test was used to identify correlates of possible PTSD. We quantified the association between correlates and PTSD by using odds ratios (ORs) and 95% confidence intervals (CIs). PTSD symptoms of surviving HCWs diagnosed with possible PTSD were described by using frequencies and percentages. The statistical significance level was set at *P* < 0.05 (two-sided). SPSS software version 12.0 package was used for analyses.

## Results

The average age of the 291 surviving HCWs was 37.3 years (standard deviation [SD]: 8.9, range: 22–73), 81.1% were women, 58.4% were nurses, and 100% reported suffering from COVID-19 as the main traumatic event they experienced. Detailed characteristics of the sample of surviving HCWs are shown in Table 1.

**Table 1:** Demographic and clinical characteristics of Chinese frontline healthcare workers who survived COVID-19 and prevalence rates of possible PTSD according to demographic, clinical, and COVID-19 exposure characteristics.

Characteristics		*n*	Number of subjects with possible PTSD (%)	*x*^2^	*P*
*Demographics*					
Gender	Male	55	8 (14.5)		
	Female	236	50 (21.2)	1.233	0.267
Age (years)	18–34	128	26 (20.3)		
	35–49	132	24 (18.2)		
	50+	31	8 (25.8)	0.935	0.626
Education	Associate degree and below	70	19 (27.1)		
	Bachelor	182	34 (18.7)		
	Master and above	39	5 (12.8)	3.695	0.158
Years of work experience	<5	34	6 (17.6)		
	5–10	86	14 (16.3)		
	>10	170	38 (22.4)	1.450	0.484
Type of workers	Doctors	63	7 (11.1)		
	Nurses	170	39 (22.9)		
	Others	58	12 (20.7)	4.057	0.132
*Clinical characteristics*					
Severity of COVID-19 during the acute period	Mild and moderate	100	16 (16.0)		
	Severe and critical	191	42 (22.0)	1.475	0.224
Number of respiratory symptoms during the most symptomatic period	≤10	240	36 (15.0)		
	>10	51	22 (43.1)	20.867	<0.001
Length of hospital stay (days)	≤16	151	25 (16.6)		
	>16	140	33 (23.6)	2.240	0.134
ICU admission	No	276	46 (16.7)		
	Yes	15	12 (80.0)	35.758	<0.001
*Residual symptoms of COVID-19*					
Headache	No	191	27 (14.1)		
	Yes	100	31 (31.0)	11.697	0.001
Dizziness	No	203	27 (13.3)		
	Yes	88	31 (35.2)	18.494	<0.001
Palpitation	No	153	23 (15.0)		
	Yes	138	35 (25.4)	4.851	0.028
Difficult breathing	No	217	32 (14.7)		
	Yes	74	26 (35.1)	14.374	<0.001
*Exposure to COVID-19*					
Physical harm	No	48	4 (8.3)		
	Yes	243	54 (22.2)	4.845	0.028
Life in danger	No	70	1 (1.4)		
	Yes	221	57 (25.8)	19.773	<0.001
Physical harm of others	No	113	15 (13.3)		
	Yes	178	43 (24.2)	5.130	0.024
Life in danger of someone else	No	80	6 (7.5)		
	Yes	211	52 (24.6)	10.684	0.001
Other traumatic events in addition to COVID-19	No	259	47 (18.1)		
	Yes	32	11 (34.4)	4.700	0.030

The average age of the 42 controls was 36.0 years (SD: 6.8), and 83.3% were women. The control group and survivor sample were comparable in terms of age (*t* = 1.055, *P* = 0.295) and gender (*χ*^2^ = 0.121, *P* = 0.728).

Fifty-eight surviving HCWs had possible PTSD, while 2 controls had possible PTSD. The rates of possible PTSD were significantly higher in surviving HCWs than in controls (19.9% vs. 4.8%, *P* = 0.017).

Results of the Chi-square test show that survivors with possible PTSD were significantly more likely to have more than 10 respiratory symptoms during the acute phase, to have been admitted to ICU, to be physically harmed by COVID-19, to have their lives in danger due to COVID-19, to witness others physically harmed by COVID-19, to witness others’ lives in danger due to COVID-19, and to be exposed to other traumatic events in addition to COVID-19. In addition, they were more likely to suffer residual symptoms of headache, dizziness, palpitations, and difficult breathing (*P* < 0.05) (Table 1).

Multiple logistic regression analysis revealed that possible PTSD was significantly associated with ICU admission (OR = 8.73, *P* = 0.003), >10 respiratory symptoms during the most symptomatic period of COVID-19 (OR = 3.08, *P* = 0.006), the residual symptom of dizziness (OR = 2.43, *P* = 0.013), the residual symptom of difficult breathing (OR = 2.23, *P* = 0.027), life in danger due to COVID-19 (OR = 16.59, *P* = 0.006), and exposure to other traumatic events (OR = 2.94, *P* = 0.035) (Table 2).

**Table 2:** Results of multiple logistic regression analysis on factors associated with possible PTSD in Chinese frontline healthcare workers who survived COVID-19.

Characteristics		OR (95%CI)	*P*
ICU admission	No	1	
	Yes	8.73 (2.11, 36.18)	0.003
Number of respiratory symptoms during the most symptomatic period of COVID-19	≤10	1	
	>10	3.08 (1.38, 6.86)	0.006
Residual symptom of dizziness	No	1	
	Yes	2.43 (1.21, 4.87)	0.013
Residual symptom of difficult breathing	No	1	
	Yes	2.23 (1.09, 4.55)	0.027
Life in danger	No	1	
	Yes	16.59 (2.20, 125.19)	0.006
Other traumatic events in addition to COVID-19	No	1	
	Yes	2.94 (1.08, 8.02)	0.035

Table 3 displays the PTSD symptoms in survivors with possible PTSD. The most common symptoms of intrusion, avoidance, and hyperarousal clusters were feeling emotionally upset when reminded of the event (91.4%), trying not to think or talk about the event (84.5%), and having trouble concentrating (84.5%), respectively. Less common symptoms were having nightmares about the event (34.5%), suddenly feeling like they were living through the event suddenly (25.9%), being unable to remember an important part of the event (32.8%), and overalertness (31.0%).

**Table 3:** PTSD symptoms of Chinese surviving frontline healthcare workers with possible PTSD (*n* = 58).

Symptoms	*n* (often or very often)	%
*Intrusion*		
Did the event cause upsetting thoughts or images that came to your mind although you didn’t want them to?	52	89.7
Did you have nightmares about the event?	20	34.5
Has it ever happen to you that you felt like living through the event suddenly again?	15	25.9
Did you feel emotionally upset when you were reminded of the event (feeling helpless, angry, sad, guilty)?	53	91.4
Did you have physical reactions when you were reminded of the event (e.g., uneasiness, shiver, or fast heartbeat)?	29	50.0
*Avoidance*		
Have you tried not to think about the event, not to talk about it, or to suppress feelings about it?	49	84.5
Did you try to avoid situations that remind you of the event (e.g., activities, people, or places)?	33	56.9
Were you unable to remember an important part of the event?	19	32.8
Did you lose interest in activities which had been important for you before the event took place (e.g., hobbies, sport)	46	79.3
Did you feel alienated or isolated from people in your environment?	41	70.7
Did you feel emotional numb (e.g., being unable to cry or unable to have positive feelings)?	32	55.2
Did you feel like your plans for the future and hopes will not come true (e.g., to start a family, less luck in life or in business than the others)?	35	60.3
*Hyperarousal*		
Did you have trouble falling or staying asleep?	48	82.8
Did you have fit of rages or were you often nervous?	45	77.6
Did you have trouble concentrating (e.g., forgetting what you just wanted to do, or forgetting what you just read or what you saw on television)?	49	84.5
Were you overly alert (e.g., checking to see who is around you, having a phone close-by for calling help if it was necessary)?	18	31.0
Were you easily startled or highly nervous (e.g., by loud noises)?	39	67.2

## Discussion

To the best of our knowledge, this is the first study in China to examine the clinical epidemiology of possible PTSD in frontline HCWs who survived COVID-19 6 months after the COVID-19 outbreak. The main finding was the 19.9% prevalence of possible PTSD in surviving HCWs, which was four times as high as that in HCWs who were free from COVID-19. Second, factors significantly associated with possible PTSD were, in order of importance, life in danger due to COVID-19, ICU admission, >10 respiratory symptoms during the most symptomatic period of COVID-19, exposure to other traumatic events, the residual symptom of dizziness, and the residual symptom of difficult breathing. Third, in comparison to typical PTSD symptoms in DSM-IV-TR criteria, recurrent distressing dreams of the event, feeling as if the traumatic event was recurring (“flashbacks”), inability to recall an important aspect of the trauma, and overalertness were less common.

In the Chinese general population, the lifetime, 12-month, and 1-month prevalence rates of PTSD were 0.30%, 0.20%, and 0.195%, respectively [25,26,]. Compared to these rates and the 4.8% prevalence of possible PTSD in the control group of this study, we found a much higher prevalence of possible PTSD in surviving HCWs. This extraordinarily high relative risk of possible PTSD in surviving HCWs is expected because of their past “double exposure” to COVID-19, as they directly suffered from the disease and its traumatic treatment and they witnessed patients’ suffering and deaths [18]. It is noteworthy that this high risk of possible PTSD in surviving HCWs was found 6 months after the acute exposure to COVID-19, possibly suggesting the chronic course of PTSD. Based on our interview experiences with these survivors, the other possible reason for this phenomenon is no timely specialized mental health services were provided to this population until our mental health care scheme, including diagnosis, psychotherapy, and psychopharmacotherapy. There is convincing evidence that persons with untreated PTSD are more likely to have a prolonged disease course [27].

In both the general population and HCWs, females are at significantly higher risk of PTSD than males [10,28,]. However, the higher prevalence of possible PTSD in females than males in our sample (21.2% vs. 14.5%) did not reach the level of statistical significance. We speculate that this would be due to the small sample size of our study, particularly male participants. Evidence shows that trauma factors are significant determinants of the development of PTSD, including the severity of trauma and number of traumatic events [28,29,]; for example, people who are directly exposed to trauma, have a perceived fear of death when experiencing the trauma, and have multiple traumatic experiences are more likely to develop PTSD. Accordingly, life in danger due to COVID-19 and exposure to other traumatic events in addition to COVID-19 were found to be significant correlates of possible PTSD in the present study. For similar reasons, for example, the total number of respiratory symptoms during the most symptomatic period of COVID-19 may reflect the degree of patients’ suffering from COVID-19, the association of possible PTSD with >10 respiratory symptoms during the most symptomatic period of COVID-19 is expected. We do not think that residual symptoms of dizziness and difficult breathing would result in PTSD; nevertheless, it is likely that the occurrence of the two respiratory symptoms exacerbates some PTSD symptoms. For example, survivors with dizziness and difficult breathing are more likely to have difficulties in falling asleep and concentrating, thereby meeting DSM-IV-TR criteria for hyperarousal in PTSD.

Empirical data suggest that ~1/4 of patients who stay in the ICU would meet the diagnostic criteria for PTSD [30]. Similarly, ICU admission was a significant correlate of possible PTSD in surviving HCWs in our study, which may be explained by the trauma caused by invasive medical procedures in the ICU such as tracheotomy and mechanical ventilation, as well as by confronting with their own mortality.

In general, PTSD symptoms vary from individual to individual, but a combination of flashbacks and nightmares, avoiding reminders of the traumatic event, emotional numbing, and increased arousal are typical symptoms [31]. In our study, most PTSD symptoms according to DSM-IV-TR criteria were presented in surviving HCWs with possible PTSD, but nightmares of the event, flashbacks, amnesia of important aspects of trauma, and overalertness were less common. This symptom pattern may be related to the nature of the traumatic event, COVID-19, which is a life-threatening infectious disease, unlike other common traumas such as earthquakes and traffic accidents. For example, persons with earthquake-induced PTSD are more likely to have nightmares about the earthquake and to suddenly feel like they are living through the event because this trauma occurs in a horrible, suddenly, and disastrous manner and may shock them. The relatively low incidence of COVID-19-related nightmares is in line with our clinical experiences from individual survivors; nevertheless, survivors with possible PTSD still reported a variety of nightmares other than COVID-19-related nightmares, such as the rushing down of mountain torrents, earth crumblings, and monster raids. We speculate that these nightmares might be triggered by COVID-19, and this interesting phenomenon deserves further clinical attention.

This study has several limitations. First, due to logistical reasons, we made the diagnosis of PTSD provisionally, based on the self-report ETI, and not through face-to-face interviews by mental health specialists. There might be some false-positive cases in the detected patients. However, because surviving HCWs and controls were assessed with the same diagnostic algorithm, the significantly higher risk of PTSD in survivors is convincing. Second, our sample of surviving HCWs was composed of volunteers; therefore, the generalizability of the study findings might be limited. Third, the sample size of this study is relatively small, also limiting the generalizability of our findings. Fourth, the data of this study were collected cross-sectionally, so causality between identified correlates and possible PTSD cannot be ascertained. Finally, surviving HCWs’ attitudes towards mental health services are potentially important for the health service delivery because we found that some PTSD survivors were reluctant to receive psychiatric treatment. Unfortunately, this study did not assess their attitudes, which is a limitation to be addressed in future studies.

In summary, possible PTSD is very prevalent among Chinese frontline HCWs who survived COVID-19 6 months after the COVID-19 outbreak, indicating the high mental health care needs of this population. There is an urgent need for mental health workers to address the epidemic of PTSD in surviving HCWs. Amongst surviving HCWs, a range of factors, in particular traumatic experiences and ICU admission, were associated with PTSD. In efforts to prevent or reduce the incidence of s PTSD in surviving HCWs, it may be useful to target those whose lives were in danger due to COVID-19, those who were admitted to the ICU, those with more respiratory symptoms during the most symptomatic period of COVID-19, those with exposure to other traumatic events, and those with residual symptoms of dizziness and difficult breathing. Mental health workers need to be cautious when assessing the presence of PTSD in this population, because of their symptom pattern, such as less commonly seen nightmares of the event, flashbacks, amnesia of important aspects of trauma, and overalertness. Mental health services for frontline HCWs who survived COVID-19 should include periodic screening for PTSD and other mental health problems to ensure early detection and treatment; expanded social supports that specifically focus on improving mental wellbeing; and, when necessary, psychiatric assessment, psychotherapy, and psychopharmacological treatment. In addition, mental health education should also be provided, because it is potentially important for increasing survivors’ adherence to mental health services and necessary psychiatric treatment. Given that the provision of mental health services is inadequate in China, hospital managers and administrators, including heads of clinical departments, should have an active role in preventing and reducing mental health problems of surviving HCWs. For example, hospital managers and administrators could provide expanded social support, mental health education, and periodic assessment of mental health and engage in follow-up care.
